# Improving low-cost inertial-measurement-unit (IMU)-based motion tracking accuracy for a biomorphic hyper-redundant snake robot

**DOI:** 10.1186/s40638-017-0069-z

**Published:** 2017-11-10

**Authors:** Weixin Yang, Alexandr Bajenov, Yantao Shen

**Affiliations:** Electrical and Biomedical Engineering, University of Nevada, Reno, N. Virginia St, Reno, NV 89557 USA

**Keywords:** Snake robot, 3D printing, IMU, Motion tracking

## Abstract

This paper develops and experimentally validates a 3D-printed snake robot prototype. Its structure is designed to allocate limited room for each functional module (including an external power module, battery power module, the wireless control and transmission module and some detective sensors), so as to ensure the snake robot works in different environments. In order to control and track the snake robot, a low-cost MEMS-IMU (micro-electro-mechanical systems inertial measurement unit)-based snake robot motion tracking system is developed. Three algorithms (low-pass filter, baseline calibration, and Kalman filter) are used to eliminate noise from IMU’s acceleration data, thus minimizing the noise influence to tracking accuracy. Through signal processing, the IMU acceleration data can be effectively used for motion tracking. The result from the video tracking software is employed as a reference for comparison, so as to evaluate the motion tracking algorithm efficiency. The comparison results demonstrate high efficiency of the proposed IMU-based motion tracking algorithm.

## Background

Biological snake has a cord-like body with many links (bones) which can form diverse locomotion modes with many functions [1]. Snake is one of the most successfully evolved species, which has lived on the earth for 300 million years because of its high adaptation to the environment. Different locomotion modes also have various functions. Some snakes have the ability to change their movement mode immediately when entering into a new environment. Because of the snake’s versatility, the study of snake locomotion mode promises many future applications such as space exploration, environmental monitoring, transportation, and surveillance.

According to the research on the biological snake, the snake-inspired robot has extremely high redundancy with many degrees of freedom, with its body on the ground and kinetically stable. This feature makes the snake easily adapt to irregular terrain. Based on this high adaptability, the snake robot can be adopted where human beings cannot go to carry out tasks. The snake’s locomotion modes can be categorized into following four types: (1) serpentine locomotion; (2) rectilinear locomotion; (3) rolling locomotion; and (4) sidewinding locomotion. In this paper, two locomotion modes are developed. The serpentine movement is the most typical locomotion mode, which has been widely observed in almost all species of snakes. According to Hirose’s [2] research on the biological snake, the research starts with generating the serpentine curve for the snake robot [3]. To test additional locomotion performance of the robot, sidewinding is further investigated and performed on the developed robot.

The snake robot is designed to collect information in some challenging environments, such as forest, cave, building ruins, or even underwater. Most of these environments are GPS-denied, leading to the difficulties in tracking and controlling snake robot. Hence, it is necessary to consider and understand the efficiency of each locomotion mode in different environments based on collected terrain information. Many studies on object’s attitude determination with MEMS-IMU have been performed for many years. MEMS-IMU-based tracking technology [4] is widely applied in many fields, such as detection of unconstrained walking [5], pedestrian tracking in indoor environments [6, 7].

However, IMU-based tracking system calculates the position by double integration of measured acceleration, and with double integration, calculation errors grow rapidly, thus the system being not reliable for a long-time tracking without the aid of GPS or another reference system. Hence, this research focuses on accuracy of IMU-based tracking system by using an improving acceleration estimation method.

To eliminate the effects of accelerometer errors, most of the papers adopt some sensor fusion algorithms, among which the Kalman filter is the most commonly used. In [8–10], standard Kalman filter, unscented Kalman filter (UKF), and extended Kalman filter (EKF) are employed in high data rate signal processing. EKF has lower accuracy; however, UKF requires more computational time.

The paper is organized as follows. In "Custom-built snake robot" section, a customized snake robot is built through 3D printing. "Snake robot trajectory tracking" section presents the motion tracking algorithm along with the three signal processing algorithms including Butterworth low-pass filter, baseline calibration, and Kalman filter. After data processing, the acceleration data can be used for motion tracking. The conclusion and discussion are given in "Conclusion" section.

## Custom-built snake robot

**Fig. 1:**
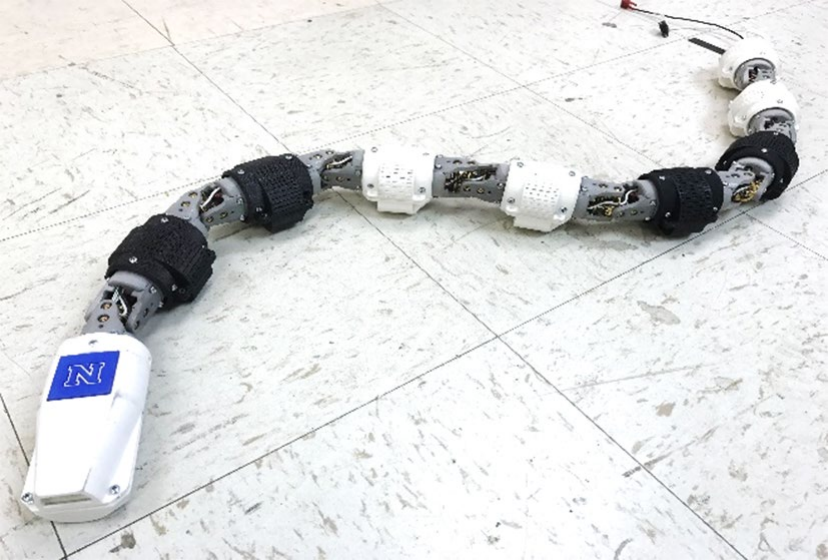
3D printed snake robot

The snake robot joint structure is modularly designed, with the upper part and the lower part being designed separately. Therefore, different chassis can be used based on the environment. The snake robot is composed of eight identical modular units. The joint model size is 50 mm in diameter and 144 mm in length. The volume of the snake robot’s body is widened by increasing the body width, and thus the integrated electronic system can be installed for controlling. This snake robot has two different bending axes connected alternately at 90 $^{\circ }$C. This allows the snake robot to have 3D motion because of the 2 degrees of freedom for each joint (total 16 degrees of freedom). Moreover, each joint can be controlled separately. The hollowed-out grids structure on robot’s body increases the structure flexibility, thus enhancing its coefficient of restitution and making the robot run efficiently. The 3D-printed snake robot is shown in Fig. 1.

## Snake robot trajectory tracking

### Motion tracking algorithm

**Fig. 2:**
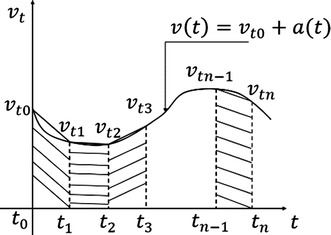
Speed–time curve [11]

Snake robot displacement can be obtained by the double integral of its acceleration. A low-cost IMU is installed in the head of the snake robot, in order to collect its real-time acceleration data at three axes with high frequency. At the same time, all acceleration data are transferred to a computer for calculation. Then the robot’s instant speed curve and accumulated space trajectory are graphed on the screen. Acceleration data are processed by MATLAB. The basic algorithm is shown in Fig. 2.

Assume sampling starts at time $t_0$. According to the integration principle, the calculation relationship among displacement *s*(*t*), velocity *v*(*t*), and acceleration *a*(*t*) in the continuous-time domain is:

1 $$\begin{aligned} s(t)= & {} \int _{t_0}^{t}{v(t)}\,\hbox {d}t+s(t_0) \end{aligned}$$

2 $$\begin{aligned} v(t)= & {} \int _{t_0}^{t}{a(t)}\,\hbox {d}t+v(t_0) \end{aligned}$$

$s(t_0)$ is accumulative displacement from 0 to $t_0$, $v(t_0)$ is the system instant motion velocity at $t_0$.

Since the outputs of the IMU are groups of discrete data, the time–speed curve is decomposed as numbers of a right-angled trapezoid. Initials$(t_0 )=0$, the displacement *s*(*t*) is presented as:

3 $$\begin{aligned} s(t)& {}= \int _{t_0}^{t}{v(t)}\,\hbox {d}t \nonumber \\& {}= \frac{v(t_0)+v(t_1)}{2}(t_1-t_0)+\frac{v(t_2)+v(t_1)}{2}(t_2-t_1)+ \cdots +\frac{v(t_n)+v(t_{n-1})}{t_n-t_{n-1}} \end{aligned}$$

$t_1-t_0=t_2-t_1=\cdots =t_n-t_{n-1}=\Delta t$, which is the sampling time interval, when $n>1,$

4 $$\begin{aligned} s(t)=\sum _{k=1}^{n}\frac{v(t_k)+v(t_{k-1})}{2}\cdot \Delta t \end{aligned}$$

In the discrete domain, the Eq. (4) is modified as:

5 $$\begin{aligned} s[n]=\sum _{k=1}^{n}\frac{v[k]+v[k-1]}{2}\cdot \Delta t \end{aligned}$$

when $n>1$, in the discrete domain

6 $$\begin{aligned} v[n]& {}= \sum _{k=1}^{n}\frac{a[k]+a[k-1]}{2}\cdot \Delta t \nonumber \\& {}= v[0]+\frac{1}{2}(a[0]+a[n])\cdot \Delta t+(a[1])+a[2]+\dots +a[n-1])\cdot \Delta t \end{aligned}$$

7 $$\begin{aligned} s[n]&= \sum _{k=1}^{n}\frac{v[k]+v[k-1]}{2}\cdot \Delta t \\&= \frac{1}{2}(v[0]+v[n])\cdot \Delta t+(v[1])+v[2]+\dots +v[n-1])\cdot \Delta t \end{aligned}$$

Then the object’s displacement in one axis is presented as:

8 $$\begin{aligned}s[n]&= n\cdot v[0]\cdot [(n-1)\cdot a[1]+(n-2)\cdot a[2] \\&\quad +\cdots +v[n-1]]\cdot \Delta t^2+\frac{1}{4} (a[0]+a[n])\cdot \Delta t^2 \end{aligned}$$

Through Eq. (8), it is easy to calculate the displacement after obtaining the initial velocity and acceleration from the IMU. However, based on this equation, the calculation is very complicated for large *n* which leads to huge burden to the system since the system needs to allocate huge memory sources to store the acceleration data from time number 0 to *n*. Furthermore, the system has to repeat all the previous acceleration calculations to update the object’s motion displacement. Thus, iteration is applied to simplify the calculation.

From Eq. (6)

9 $$v[n]-v[n-1]= \frac{a[n]+a[n-1]}{2}\cdot \Delta t$$

10 $$\begin{aligned} s[n]-s[n-1]& {}= \frac{v[n]+v[n-1]}{2}\cdot \Delta t \nonumber \\& {}= v[n-1]\cdot \Delta t+\frac{1}{4}(a[n-1]+a[n])\cdot \Delta t^2 \end{aligned}$$

Equations (9) and (10) are modified as:

11 $$\begin{aligned} v[n]& {}= v[n-1]+\frac{a[n]+a[n-1]}{2}\cdot \Delta t \end{aligned}$$

12 $$\begin{aligned} s[n]& {}= s[n-1]+\frac{v[n]+v[n-1]}{2}\cdot \Delta t \nonumber \\& {}= s[n-1]+v[n-1]\cdot \Delta t+\frac{1}{4}(a[n-1]+a[n])\cdot \Delta t^2 \end{aligned}$$

From Eqs. (11) and (12), the object’s instant speed *v*[*n*] and motion displacement *s*[*n*] can be calculated recursively. The IMU has the acceleration outputs at *X*-, *Y*- and *Z*- axis. Based on Eq. (11), the object’s instant speed at *X*-, *Y*- and *Z*-axis can be presented as:

13 $$\begin{aligned} v_x[t]& {}= \bar{v_x}[t-\Delta t]+\frac{\bar{a}_x[t]+a_x[t-\Delta t]}{2}\cdot \Delta t \end{aligned}$$

14 $$\begin{aligned} v_y[t]& {}= \bar{v_y}[t-\Delta t]+\frac{\bar{a}_y[t]+a_y[t-\Delta t]}{2}\cdot \Delta t \end{aligned}$$

15 $$\begin{aligned} v_z[t]& {}= \bar{v_z}[t-\Delta t]+\frac{\bar{a}_z[t]+a_z[t-\Delta t]}{2}\cdot \Delta t \end{aligned}$$

The object’s instant speed in 3D space at time *t* can be calculated as:

16 $$\begin{aligned} v[t]& {}= \bar{v}_x[t]+\bar{v}_y[t]+\bar{v}_z[t] \end{aligned}$$

Similarly, the object’s motion displacement at *X*-, *Y*- and *Z*-axis is presented as:

17 $$\begin{aligned} s_x[t]& {}= s_x[t-\Delta t]+v_x[t-\Delta t]\cdot \Delta t+\frac{1}{4}(a_x[t-\Delta t]+a_x[T])\cdot \Delta t^2 \end{aligned}$$

18 $$\begin{aligned} s_y[t]& {}= s_y[t-\Delta t]+v_y[t-\Delta t]\cdot \Delta t+\frac{1}{4}(a_y[t-\Delta t]+a_y[T])\cdot \Delta t^2 \end{aligned}$$

19 $$\begin{aligned} s_z[t]& {}= s_z[t-\Delta t]+v_z[t-\Delta t]\cdot \Delta t+\frac{1}{4}(a_z[t-\Delta t]+a_z[T])\cdot \Delta t^2 \end{aligned}$$

During time $t-\Delta t$, the object’s space motion displacement is:

20 $$\begin{aligned} s_\Delta [t]= & {} \sqrt{(s_x[t]-s_x[t-\Delta t])^2+(s_y[t]-s_y[t-\Delta t])^2+(s_z[t]-s_z[t-\Delta t])^2} \end{aligned}$$

So at time *t*, the object’s space coordinate is $(s_x[t], s_y[t], s_z[t])$. In the space coordinate system, the object’s trajectory can be plotted by connecting all the space coordinate points from $t_0$ to $t_n$.

### Noise processing of tracking system

**Fig. 3:**
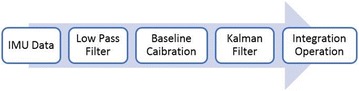
Data processing flow diagram

Since the tracking system is based on the double integral of acceleration, errors of IMU measurements cause incorrect integration results and accumulated bias during integration. Even minimal measurement errors will result in huge calculation errors, leading to the low accuracy in tracking results. This paper proposes several methods to filter the acceleration data, so as to make the acceleration data available to integrate. The flow diagram for data processing is shown in Fig. 3.

#### Low-pass filter

**Fig. 4:**
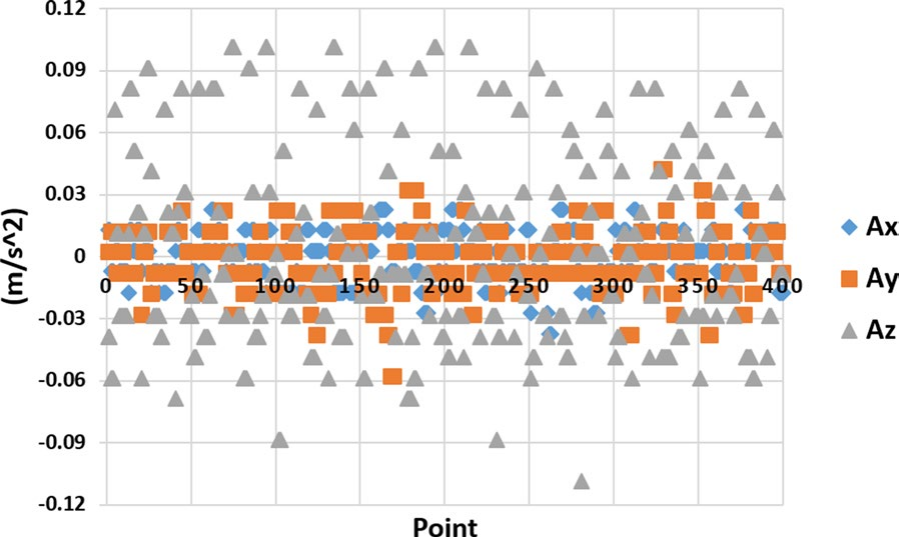
Acceleration data when stationary

IMU has noisy signals when stationary. To analyze the impact of noise, 16,000 stationary data are collected, i.e. 4000 points for each axis plus 4000 starting points. Then, calculate the average acceleration value of each axis, finally, subtracting the average value from every single data for each axis, thus getting the acceleration bias of each axis which is shown in Fig. 4.

**Fig. 5:**
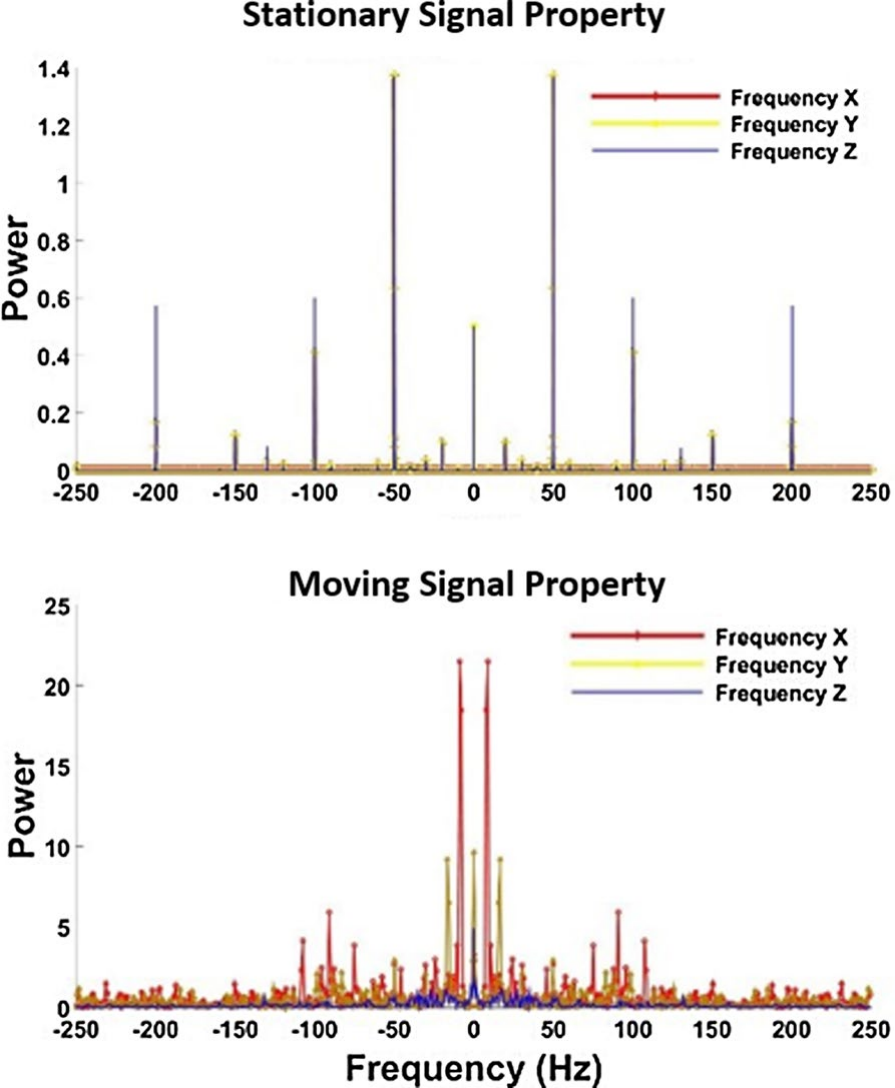
Signal properties in frequency domain

From Fig. 4, the acceleration vibrates randomly with a maximum value of 0.05 m/s$^2$. That means the object has maximum 0.05 m shift when it keeps stationary. Therefore, the errors have a great influence on the tracking system accuracy. To eliminate noise, it is necessary to analyze the signal properties in the frequency domain as shown in Fig. 5.

After Fourier transform of the acceleration data, it is clear in the above picture of Fig. 5 that the noise of each axis is mainly around 50 Hz in stationary. From the lower picture of Fig. 5, the valid acceleration data are mainly around 10 Hz for the *X*-axis and 15 Hz for the *Y*-axis when moving.

By programming IMU, the data output frequency is 50 Hz. A hardware RC low-pass filter is employed between IMU output terminal and wireless transmission chip, so as to reduce high-frequency component. Then by using Butterworth low-pass filter, most of the high-frequency noises are inhibited. The signal decay of the Butterworth low-pass filter is slow but smooth. In this paper, the sampling rate is 500 Hz; the passband corner frequency is 15 Hz; the stopband corner frequency is 50 Hz; the maximum passband ripple is 3 dB; and the minimum attenuation is 60 dB.

**Fig. 6:**
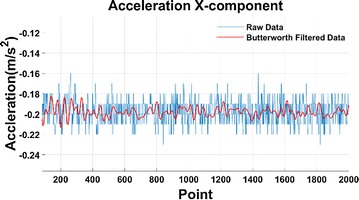
Filtered acceleration data

**Fig. 7:**
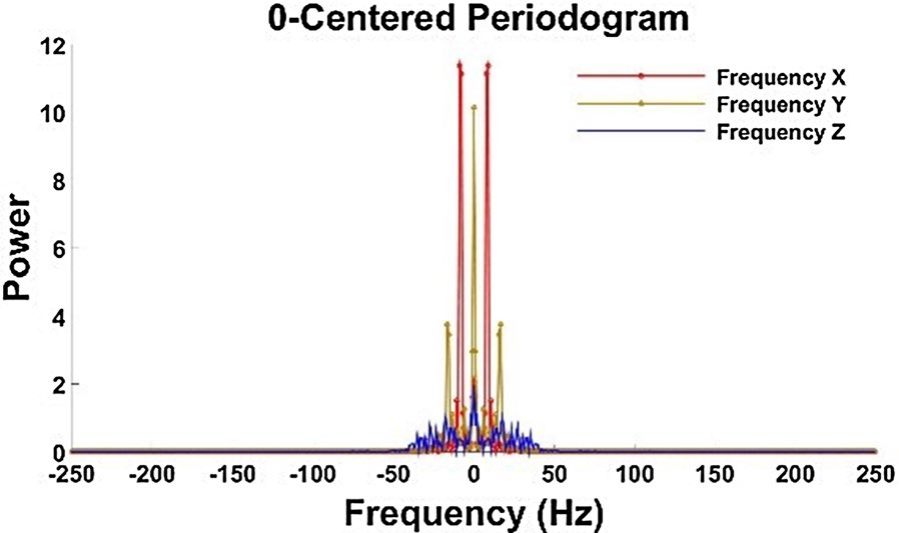
Spectrogram of moving acceleration after Butterworth low-pass filter

The time-domain graph and spectrogram of acceleration after Butterworth low-pass filtering of the stationary snake robot are shown in Figs. 6 and 7 respectively.

It is clear in Fig. 7, there is noise only in the passband, and its amplitude is very small.

#### Baseline calibration

After low-pass filter, the acceleration data can be used for integration. Before integrating the data, the baseline shift cannot be ignored.

**Fig. 8:**
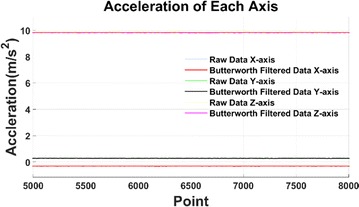
Baseline shift after moving

The snake moves forward in a line and then stays stationary. After low-pass filter, only the stationary acceleration data of each axis are collected and plotted as shown in Fig. 8.

The baseline shift in the *X*-axis is about − 0.3 m/s$^2$, i.e. the *X*-axis movement when tracking is − 0.3 m/s even if the snake robot is stationary. Similarly, the baseline shift in the *Y*-axis is about 0.3 m/s$^2$, i.e. the *Y*-axis movement is 0.3 m/s when stationary. When including the baseline shift is in the integration, the cumulative errors will have a significant influence on the system accuracy.

**Fig. 9:**
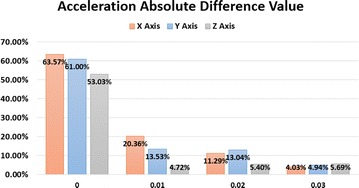
Absolute difference of acceleration when stationary

The absolute difference of acceleration presents the relative change of acceleration data. When the snake robot moves fast, the absolute difference becomes relatively big. Also, the absolute difference can present the noise oscillation when the snake robot maintains stationary. MATLAB is used to plot the absolute difference distribution of acceleration, which is shown in Fig. 9.

The above statistic shows that the data oscillation in *X*-axis and *Y*-axis is within 0.05, while in the *Z*-axis direction, the oscillation is in a wider range of 0.1. Table 1 shows the probability of absolute difference within the range when the snake robot keeps stationary.

**Table 1:** Statistic of oscillation distribution when stationary

*X*-axis < 0.05	*Y*-axis < 0.05	*Z*-axis < 0.1
1	0.9418	0.9548

The results show the big probability of the absolute difference is within 0.1 when the snake robot is stationary. To compare with the moving acceleration data, the same method is employed. The statistic of oscillation distribution in moving state is shown in Table 2.

**Table 2:** Statistical analysis of oscillation distribution when moving

*X*-axis < 0.05	*Y*-axis < 0.05	*Z*-axis < 0.1
0.0839	0.0704	0.0124

The distribution of absolute difference for motion data is concentrated on a smaller interval comparing with stationary data, which has been verified by repeated experiments.

 Putting 1000 data from *Y*-axis in one group to calculate the probability of range within [0, 0.05]. Five groups are repeated, the results of which are shown in Table 3.

**Table 3:** Statistical analysis of oscillation distribution when moving and stationary

Probability	Group 1	Group 2	Group 3	Group 4	Group 5
Stationary	0.9418	0.9854	0.9712	0.9755	0.9587
Moving	0.0704	0.0839	0.0655	0.0814	0.0716

From Table 3, the data verify the previous conclusion that the probability of the absolute difference within [0, 0.05] in stationary is much higher than that of in moving state. To calibrate the baseline, every 100 data (i.e. data in 0.2 s) are taken for the statistic. The statistic shows whether the probability of the absolute difference within [0, 0.05] is higher than 0.95. If so, then the snake robot is stationary. Then the average value of this group data is used as the new average value to shift the acceleration data, i.e., adding or subtracting the compensation value from acceleration data (depending on the sign of acceleration data). At the same time, once the data show that the snake robot is stationary, the value of acceleration data is set to zero.

**Fig. 10:**
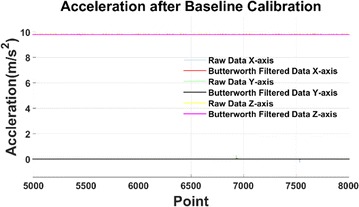
Baseline calibration result

The baseline calibration results are shown in Fig. 10. The bias after baseline calibration is zero when the snake robot is stationary.

#### Kalman filter

To eliminate random errors, the Kalman filter is applied to the tracking system, thus filtering the acceleration data. The Kalman filter is a set of mathematical equations that provide an efficient computational (recursive) state estimation, so as to minimize the mean squared error [12].

The random process to be estimated can be modeled in the form:

21 $$\begin{aligned} {x_{k+1}=\phi _kx_k+w_k+B_ku_k} \end{aligned}$$

when the process state vector is:

22 $$\begin{aligned} x_k=[Acce\_x, Acce\_y, Acce\_z] \end{aligned}$$

The initial process state vector is the initial set of acceleration data. The observation (measurement) of the process occurs at discrete points in time by the linear relationship. $\phi _k$ is the state transition matrix. $H_k$ is the measurement matrix at time $t_k$. The initial estimation error covariance is manually selected as $P_{k}^{-}$, where

23 $$\begin{aligned} z_k=H_kx_k+v_k \end{aligned}$$

$$\begin{aligned} \phi _k= \left[ \begin{array}{cccc} 1 &{} 0 &{} 0\\ 0 &{} 1 &{} 0\\ 0 &{} 0 &{} 1 \end{array} \right] H_k= \left[ \begin{array}{cccc} 1 &{} 0 &{} 0\\ 0 &{} 1 &{} 0\\ 0 &{} 0 &{} 1 \end{array} \right] P_{k}^{-}= \left[ \begin{array}{cccc} 0.05 &{} 0 &{} 0\\ 0 &{} 0.05 &{} 0\\ 0 &{} 0 &{} 0.05 \end{array} \right] \end{aligned}$$

Entering the data into standard Kalman filter, recursive loop consists of Eqs. (24), (25), (26), (27) and (28).

24 $$\begin{aligned} \hat{x}_k& {}= \hat{x}_{k}^{-}+K_k(z_k-H_k\hat{x}_{k}^{-}) \end{aligned}$$

25 $$\begin{aligned} K_k& {}= P_{k}^{-}H_{k}^{T}(H_kP_{k}^{-}H_{k}^{T}+R_k)^{-1} \end{aligned}$$

26 $$\begin{aligned} P_k& {}= (I-K_kH_k)P_{k}^{-} \end{aligned}$$

27 $$\begin{aligned} \hat{x}_{k+1}^{-}& {}= \phi _k\hat{x}_k \end{aligned}$$

28 $$\begin{aligned} P_{k+1}^{-}& {}= \phi _k P_k \phi _{k}^{T}+Q_k \end{aligned}$$

Now all the data and equations are ready for the Kalman filter loop. Once entering the loop, it will run to ad infinitum. The data are transferred by the wireless chip to the computer serial port, and then MATLAB will read the data for Kalman filter algorithm.

### Snake robot locomotion analysis

**Fig. 11:**
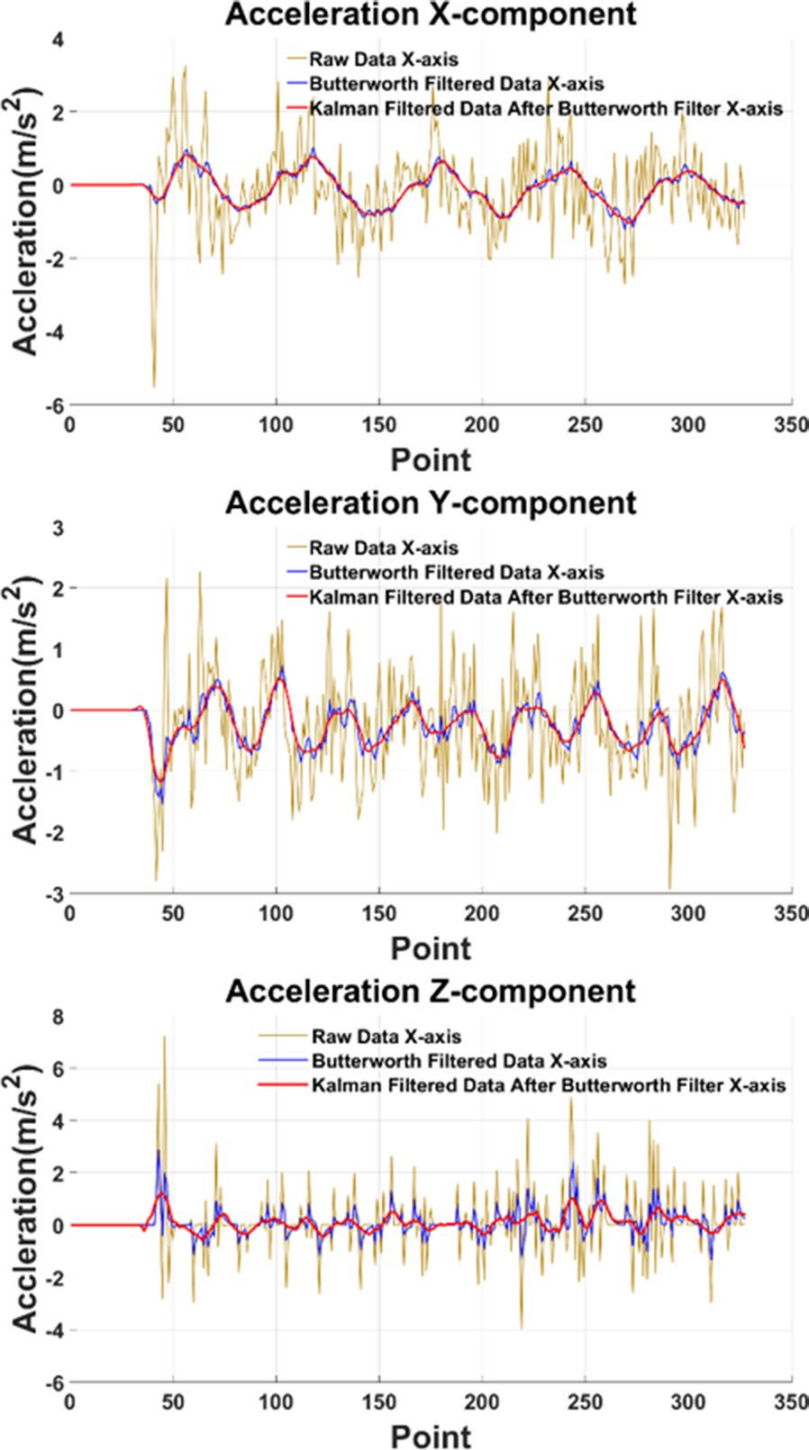
Snake robot acceleration

**Fig. 12:**
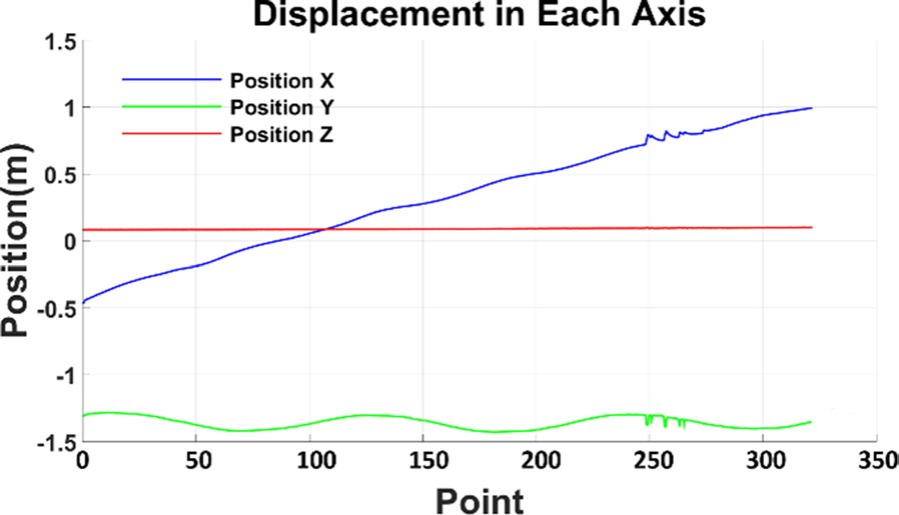
Snake robot serpentine locomotion displacement in each axis

**Fig. 13:**
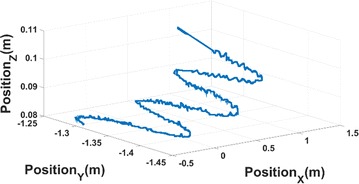
Snake robot serpentine trajectory

In the laboratory environment, IMU chip is installed in the head of the snake robot which moves along a straight line. The filtered acceleration data are shown in Fig. 11. It clearly shows in the picture that the high-frequency component of the acceleration is eliminated and the random noise is minimized. The signal curve becomes smooth and available to integrate. The IMU tracking result of snake robot’s Serpentine locomotion is shown in Fig. 12. It indicates that the serpentine locomotion has the highest efficiency because the snake robot doesn’t have slide-back either at X- or *Y*-axis. Figure 13 gives the snake robot trajectory, showing the forward locomotion in the *X* direction and sinusoidal swing in the *Y* direction. The calculated trajectory follows the real locomotion which proves the accuracy of IMU tracking system.

**Fig. 14:**
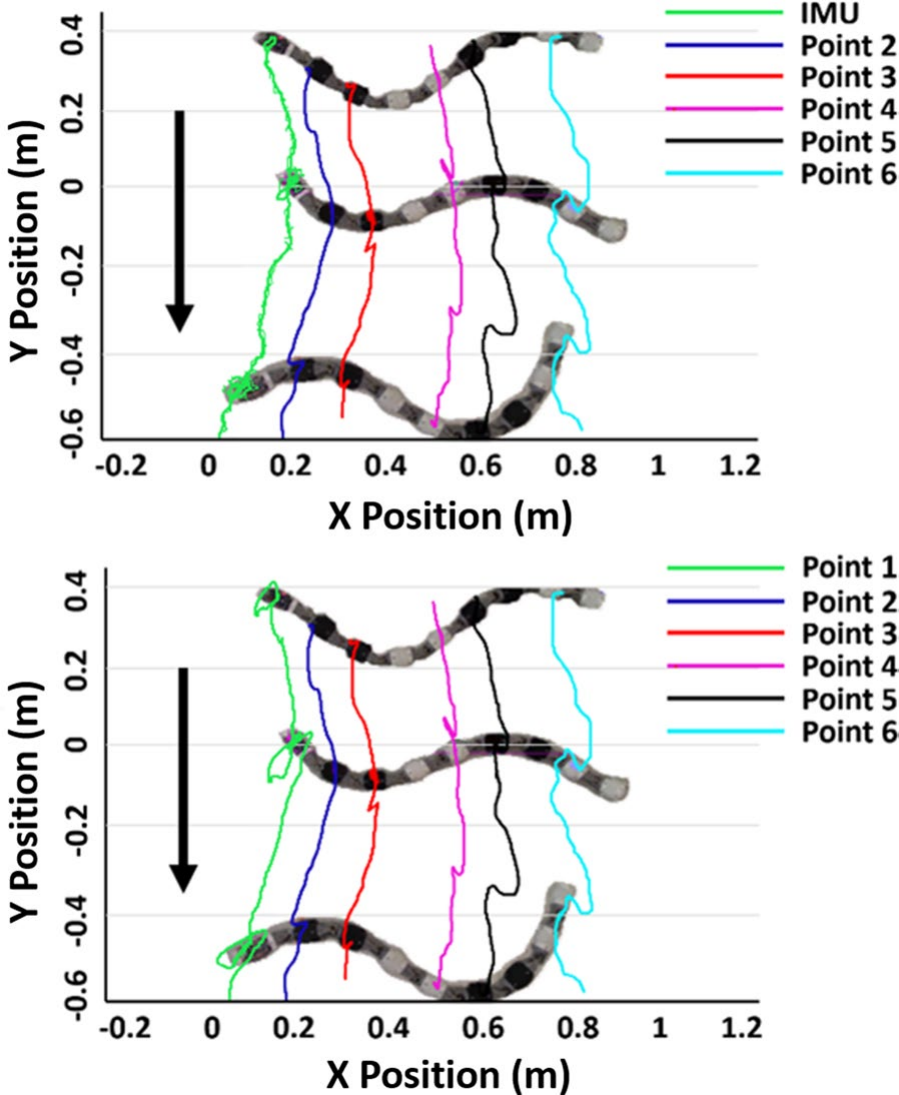
Tracking result of sidewinding locomotion

To improve snake robot adaptivity in different environments, the sidewinding locomotion mode is developed in this paper. For better locomotion analysis, it also employs a video tracking software. The 6 points video tracking results are shown in the bottom picture of Fig. 14. In the above picture, IMU tracking is used to replace video tracking of point 1 which is shown by the green line. Compared with video tracking result, IMU tracking still has some glitches, thus leading to errors. The error analysis follows.

**Fig. 15:**
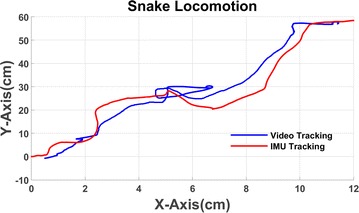
Snake robot locomotion in *XY*-plane

**Fig. 16:**
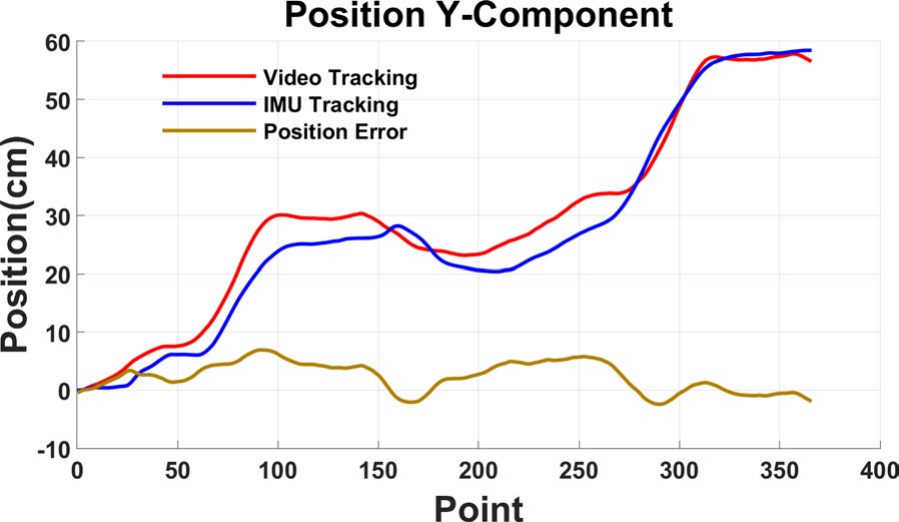
Comparison between video and IMU tracking at *Y*-axis

Let the snake robot move along a straight line. The video recorder is set to be perpendicular to the snake robot, in order to make the video tracking work well. The snake robot’s locomotion in *XY*-plane is shown in Fig. 15. Snake robot’s locomotion in *Y*-axis is used to calculate IMU tracking which is shown in Fig. 16. Comparing with the video tracking results, the IMU tracking still has small errors. The results show that the maximum error is 12.23%.

The absolute displacement and average velocity of the two tracking systems are listed in Table 4.

**Table 4:** Comparison of tracking results

	Displacement (cm)	Velocity (cm/s)
IMU tracking	106.85	28.59
Video tracking	95.25	25.34
Simulation	100	24.3

Since the video tracking software has higher accuracy, results of video tracking are used as the reference to calculate the error of IMU. Repeating the experiment to find out the average error of IMU tracking system, the results are shown in Table 5.

**Table 5:** Average tracking error from experiments

Num.	Video Dis. *X*-axis	Video Dis. *Y*-axis	IMU Dis. *X*-axis	IMU Dis. *Y*-axis	Video Ave. speed	IMU Ave. speed	Average velocity error (%)	Dis. error (%)
1	34.37	95.85	38.65	106.85	24.90	27.12	8.93	11.59
2	31.56	93.48	36.14	105.61	24.28	26.80	10.40	12.14
3	32.14	96.54	36.54	107.36	25.08	27.25	8.67	11.48
4	33.19	101.3	39.45	109.74	26.31	27.85	5.88	9.87
5	36.21	98.65	40.19	109.22	25.62	27.72	8.19	11.56
6	34.95	97.51	38.47	110.35	25.33	28.01	10.58	12.23
7	35.41	101.3	39.10	113.84	26.31	28.89	9.81	12.18
8	34.87	95.11	37.30	107.97	24.70	27.40	10.93	12.12
9	39.12	105.9	45.85	118.54	27.50	30.09	9.41	12.21
10	34.84	96.34	36.54	108.21	25.02	27.46	9.76	11.70

Based on the experiments, the average displacement error is 12.01%, and the average velocity error is 9.25%. The error may come from

- Accelerometer accuracy.
- The measurement error due to mechanical characteristics of MEMS under the influence of the environment.
- Gravity.
- Random noise.

## Conclusion

This paper presents a solution to build a snake robot with the tracking system. The tracking system/algorithm can be used for locomotion analysis based on IMU data. Experiments on the algorithm validate its effectiveness. The algorithm can be further improved as the advanced data processing methods.
